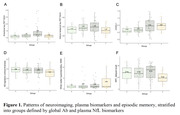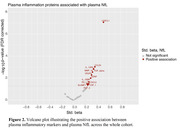# In vivo 18F‐MK6240 tau PET imaging in relation to peripheral inflammation and episodic memory in late middle‐aged adults

**DOI:** 10.1002/alz.093499

**Published:** 2025-01-09

**Authors:** Mona‐Lisa Malarte, Konstantinos Chiotis, Konstantinos Ioannou, Elena Rodriguez‐Vieitez

**Affiliations:** ^1^ Department of Neurobiology, Care Sciences and Society, Center for Alzheimer Research, Karolinska Institutet, Stockholm Sweden; ^2^ Department of Neurology, Karolinska University Hospital, Stockholm Sweden

## Abstract

**Background:**

Detecting early stages of Alzheimer's disease (AD) remains a crucial yet complex challenge. While recent interest has surged in detecting biomarkers linked with the disease preclinical phase, a comprehensive understanding of the concomitant peripheral biological pathways before the potential disease onset is necessary. We aim to explore the associations between the 18F‐MK6240 tau PET tracer with plasma inflammatory markers, other AT(X)N biomarkers and episodic memory.

**Method:**

The cross‐sectional sample consisted of n=132 cognitively unimpaired late middle‐aged Hispanic individuals (64.5 ± 3.4 years old, 34.1% APOE4 carriers, 69.7% female, 10.7 ± 4.0 years of education) from Northern Manhattan (AD Knowledge Center database). All individuals underwent multi‐modal imaging with T1‐weighted MRI, T2‐weighted FLAIR MRI, Aβ PET (18F‐florbetaben), tau PET (18F‐MK6240), plasma measures of inflammation (OLINK panel of 92 markers), plasma neuronal injury (NfL), and neuropsychological assessments. Data on demographics: age, sex, education, APOE, body mass index (BMI), and diabetes mellitus, were also included. Using the median split to dichotomize global Aβ and NfL measures, the study sample was stratified into four groups: “Aβ‐low, NfL‐low” (Group 0); “Aβ‐high, NfL‐low” (Group 1); “Aβ‐high, NfL‐high” (Group 2); and “Aβ‐low, NfL‐high” (Group 3).

**Result:**

Our results revealed that 18F‐MK6240 tau PET is sensitive in detecting early neocortical tau pathology, even without evident neuronal injury (Figure 1). Plasma inflammation was not directly linked with Aβ or tau pathologies but strongly associated with NfL (Figure 2). Markers of neuronal injury (NfL), cerebrovascular injury (WMH) and specific inflammatory markers were associated with lower episodic memory (Figure 1). High NfL correlated with factors such as high inflammation, BMI, diabetes and older age.

**Conclusion:**

The study emphasizes the complex nature of early AD markers suggesting that, aside from Aβ and tau, peripheral markers of NfL and inflammation are also central. Our findings call for a broader perspective in identifying early AD markers, underscoring the interplay between neuronal damage, inflammation, comorbidities and early cognitive changes.